# Genetic Determinants of Severe Hypertriglyceridemia: Rare Variants in *LPL*, *APOC2*, *APOA5*, *GPIHBP1*, *LMF1*, *APOE* and Polygenic Risk

**DOI:** 10.3390/ijms27125443

**Published:** 2026-06-16

**Authors:** Anastasia V. Blokhina, Alexey N. Meshkov, Alexandra I. Ershova, Marija Zaicenoka, Viktoria I. Mikhailina, Stepan A. Smetnev, Anna A. Bukaeva, Alena. S. Limonova, Anna V. Kiseleva, Evgeniia A. Sotnikova, Anastasia A. Zharikova, Elizaveta A. Novokhatskaya, Elizaveta V. Baranovskaya, Yuri V. Vyatkin, Vasily E. Ramensky, Maria S. Pokrovskaya, Oxana M. Drapkina

**Affiliations:** 1 National Medical Research Center for Therapy and Preventive Medicine, Ministry of Healthcare of the Russian Federation, Petroverigsky per. 10, Bld. 3, 101000 Moscow, Russia; meshkov@lipidclinic.ru (A.N.M.); alersh@mail.ru (A.I.E.); marija.zaicenoka@gmail.com (M.Z.); doctor031193@gmail.com (V.I.M.); stefancom@mail.ru (S.A.S.); annbukaeva@gmail.com (A.A.B.); limonova-alena@yandex.ru (A.S.L.); sotnikova.evgeniya@gmail.com (E.A.S.); azharikova89@gmail.com (A.A.Z.); lisabet244@gmail.com (E.A.N.); elizaveta.baranovskaya8@gmail.com (E.V.B.); vyatkin@gmail.com (Y.V.V.); ramensky@gmail.com (V.E.R.); mpokrovskaya@gnicpm.ru (M.S.P.); drapkina@bk.ru (O.M.D.); 2 Department of General and Medical Genetics, Pirogov Russian National Research Medical University, 1, Ostrovityanova Str., 117997 Moscow, Russia; 3 Faculty of Bioengineering and Bioinformatics, Lomonosov Moscow State University, 1-73, Leninskie Gory, 119991 Moscow, Russia; 4 Department of Natural Sciences, Novosibirsk State University, 1, Pirogova Str., 630090 Novosibirsk, Russia; 5 Institute for Artificial Intelligence, Lomonosov Moscow State University, 1-73, Leninskie Gory, 119991 Moscow, Russia

**Keywords:** severe hypertriglyceridemia, *LPL*, APOC2, *APOA5*, *GPIHBP1*, *LMF1*, *APOE*, chylomicronemia, familial dysbetalipoproteinemia, polygenic risk score

## Abstract

Severe hypertriglyceridemia (HTG) is genetically heterogeneous, but its genetic architecture remains incompletely characterized. We investigated the genetic determinants of severe HTG in 123 patients with triglyceride (TG) levels > 5.0 mmol/L and available NGS data. We analyzed rare variants in *LPL*, *APOC2*, *APOA5*, *GPIHBP1*, *LMF*1, and *APOE*; the ε2/ε2 *APOE* genotype; and TG-polygenic risk score (PRS) based on 40 variants. Major genetic determinants were identified in 65.0% of individuals, including rare variants in chylomicronemia genes (24.4%; 28 variants, including 10 novel, 53.6% in *LPL*), rare *APOE* variants or the ε2/ε2 genotype (18.7%, overlapping with chylomicronemia variants in 4.1%), and an extreme polygenic burden (35.8%; PRS > 90th percentile), including 26.0% with isolated polygenic HTG. The remaining 35.0% had moderate-to-low PRS. The cohort was categorized into familial chylomicronemia syndrome (FCS, *n* = 7), multifactorial chylomicronemia syndrome (MCS, *n* = 21), polygenic HTG (*n* = 32), familial dysbetalipoproteinemia (FD, *n* = 20), and moderate-to-low PRS (*n* = 43) groups based on genetic determinants. FCS had the lowest PRS percentile (median 26) and the most distinct clinical profile, with the highest TG levels (median 30.60 mmol/L) and 6–24-fold higher odds of pancreatitis compared with other groups (*p* < 0.05), alongside a lower body mass index (median 23.0 kg/m^2^) than all groups except MCS, whereas FD had the lowest TG levels (10.20 mmol/L, *p* < 0.05). These results further advance the understanding of the complex genetic architecture of severe HTG and demonstrate that broader genetic analysis, including *APOE* and TG-PRS, may increase the yield of genetic determinants in severe HTG.

## 1. Introduction

Hypertriglyceridemia (HTG) is a common dyslipidemia, typically defined by a fasting triglyceride (TG) level > 1.7 mmol/L, with levels > 5.65 mmol/L classified as severe HTG and levels > 10.0 mmol/L as extreme HTG [1,2], which are usually associated with chylomicronemia [3]. HTG is increasingly viewed not as a discrete diagnostic threshold, but as a continuum of metabolic dysregulation affecting the production, lipolysis, and clearance of TG-rich lipoproteins, including chylomicrons, very-low-density lipoproteins, and cholesterol-enriched remnants [2,4].

Genetic and epidemiological evidence consistently links TG-rich lipoproteins and their remnants to an increased risk of atherosclerotic cardiovascular disease, independent of low-density lipoproteins [5,6,7]. Similarly, the risk of pancreatitis increases progressively with TG levels, becoming detectable at 2.0–10.0 mmol/L, rising markedly above 10.0 mmol/L, and reaching extreme levels at ≥20.0 mmol/L [8,9,10].

At the genetic level, HTG is heterogeneous. Familial chylomicronemia syndrome (FCS), a rare cause of HTG, is driven by biallelic (homozygous or compound heterozygous) or, less commonly, double-heterozygous pathogenic variants in key genes of the lipoprotein lipase pathway, including *LPL*, *APOC2*, *APOA5*, *GPIHBP1*, and *LMF1*. FCS is typically early-onset, characterized by extreme HTG in the absence of secondary factors, and associated with a high risk of recurrent pancreatitis [3,11,12]. In contrast, multifactorial chylomicronemia syndrome (MCS) is far more common and reflects partial impairment of lipoprotein lipase activity. It may result from a single heterozygous variant, the combined effect of a rare pathogenic variant and additional variants of uncertain significance (VUS), or the cumulative impact of multiple VUSs in chylomicronemia genes [3]. MCS typically manifests in adulthood and is often accompanied by secondary factors [3,11].

Severe HTG may also be driven by rare *APOE* variants [13,14,15] or the ε2/ε2 genotype [16,17,18], which are associated with familial dysbetalipoproteinemia (FD), also known as type III hyperlipoproteinemia. FD is characterized by the accumulation of highly atherogenic, cholesterol-enriched remnants [19,20,21] and is one of the most common inherited dyslipidemias [22,23,24].

However, most cases of severe HTG in adults are likely polygenic, reflecting the cumulative effect of multiple TG-raising variants along with non-genetic factors. Polygenic HTG may also coexist with other genetic forms of HTG [25,26,27,28,29,30].

Most previous studies of severe HTG have focused on canonical chylomicronemia genes, with limited evaluation of polygenic risk [25,26,27,31,32,33,34,35]. Moreover, a comprehensive genetic characterization of severe HTG has not been performed in Eastern European cohorts. This study aimed to investigate the genetic determinants of severe HTG by jointly analyzing variants in five canonical chylomicronemia genes, *APOE* (including the ε2/ε2 genotype), as well as the TG-polygenic risk score (PRS). We further compared clinical characteristics and PRS percentiles across genetically defined groups.

## 2. Results

### 2.1. Characteristics of Study Subjects

A total of 123 index patients with TG levels > 5.0 mmol/L and available genetic data were included in the study (Section 4.1). Clinical and demographic characteristics are presented in Table 1.

The median age of the participants was 48 years (41; 54), and 59.3% were men. Most patients (72.4%) had extremely elevated TG levels (>10.0 mmol/L).

The cohort demonstrated a high burden of cardiometabolic disease. The majority of patients (86.0%) had a body mass index (BMI) > 24.9 kg/m^2^, and 42.1% were obese. Type 2 diabetes was present in 33.3% of the participants, while impaired fasting glucose or impaired glucose tolerance was observed in an additional 4.9%. The prevalence of metabolic syndrome components was 35.0%.

A history of pancreatitis was reported in 25.9% of patients. Coronary artery disease (CAD) was present in approximately one quarter of patients with severe HTG, and among these patients, 65.5% had early-onset CAD.

### 2.2. Rare Variants in Chylomicronemia Genes and APOE and the ε2/ε2 Genotype

**Figure 1:**
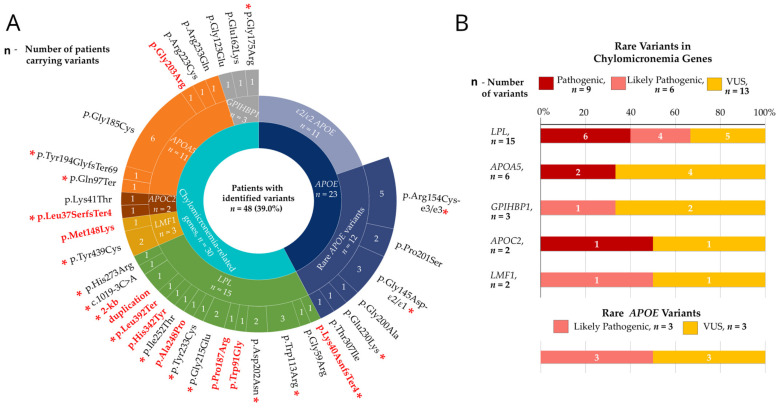
Genetic spectrum of rare variants in chylomicronemia genes and *APOE* and the ε2/ε2 genotype: (**A**) The inner rings represent patients carrying variants in five chylomicronemia genes and those with rare variants in *APOE* or the ε2/ε2 genotype. The outer ring indicates the number of patients carrying each variant. Because some patients had multiple genetic findings, the sum of variant-specific carriers exceeds the total number of affected individuals. Novel variants (*n* = 10) are highlighted in red. * indicates pathogenic or likely pathogenic variants. (**B**) Distribution of rare variants across chylomicronemia genes and *APOE*, stratified by pathogenicity. VUS—variant of uncertain significance.

Genetic data from 123 patients were analyzed for rare variants in five canonical chylomicronemia genes (*LPL*, *APOC2*, *APOA5*, *GPIHBP1*, and *LMF1*) and *APOE*. The ε2/ε2 *APOE* genotype, a major genetic determinant of FD, was also included. Overall, rare variants in these genes or the ε2/ε2 genotype were identified in 39.0% of patients (Figure 1A, Table A1 and Table A2).

#### 2.2.1. Rare Variants in Chylomicronemia Genes

Rare variants in chylomicronemia genes were identified in 30 patients (24.4%). The highest number of patients carried variants in *LPL* (12.2%), followed by *APOA5* (8.9%), *GPIHBP1* (2.4%), *LMF1* (2.4%), and *APOC2* (1.6%) (Figure 1A).

A total of 28 rare variants were identified across five genes. More than half were located in *LPL* (53.6%), followed by *APOA5* (21.4%), *GPIHBP1* (10.7%), and 7.1% each in *APOC2* and *LMF1*. Missense variants predominated (75.0%), whereas 57.1% of loss-of-function variants were found in *LPL.* Most variants (82.1%) were identified in a single index patient, while five were observed in two or more patients.

Of all identified variants, 15 (53.6%) were classified as pathogenic or likely pathogenic; of these, 66.7% were located in *LPL*. Moreover, we identified 10 previously unreported variants, including six missense VUSs, three pathogenic frameshift variants (p.Lys40AsnfsTer4 and p.Leu392Ter in *LPL*, and p.Leu37SerfsTer4 in *APOC2*), and one large-scale pathogenic duplication in *LPL* (Figure 1, Table A1).

**Figure 2:**
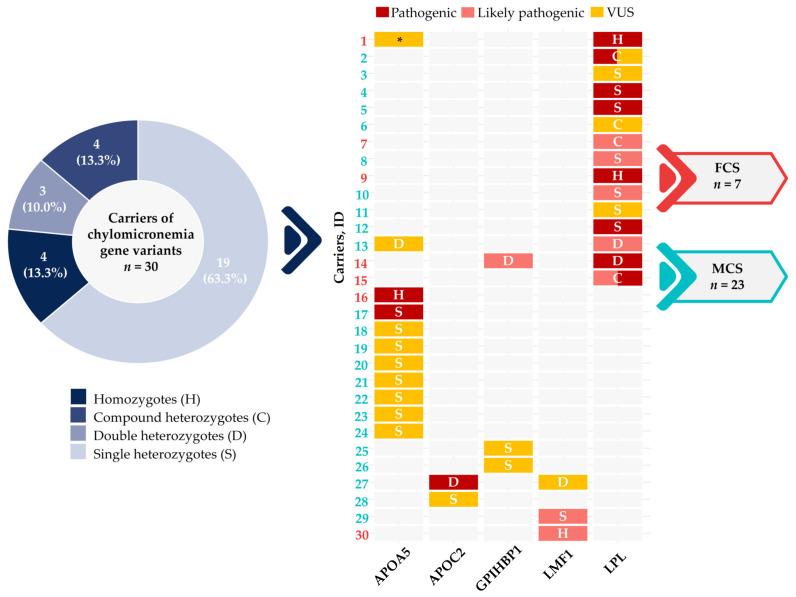
Distribution of genotypes among carriers of chylomicronemia gene variants. The donut chart shows the spectrum of genotypes. The patient–gene matrix details all 30 carriers by genes, indicating genotype and variant pathogenicity. Carrier IDs are color-coded according to the genotype-based stratification: red indicates individuals meeting genetic criteria for FCS (biallelic or double-heterozygous pathogenic or likely pathogenic variants in chylomicronemia genes), and blue indicates variant-positive MCS (a single heterozygous variant or a heterozygous pathogenic or likely pathogenic variant together with an additional VUS in chylomicronemia genes). * indicates an additional heterozygous variant in *APOA5* in a patient homozygous for a pathogenic *LPL* variant. VUS—variant of uncertain significance.

Among variant carriers, 26.6% had biallelic variants, 10.0% were double heterozygotes, and 63.3% were single heterozygotes (Figure 2).

#### 2.2.2. Genotype-Based Stratification of Variant-Positive FCS and MCS

Among the study participants, seven (5.7%) met the genetic criteria for FCS, whereas variant-positive MCS was identified in 23 of 123 patients (18.7%).

Of all FCS cases, six (85.7%) were biallelic carriers of pathogenic or likely pathogenic variants, including four homozygotes (57.1%) and two compound heterozygotes (28.6%). Biallelic variants were most frequently identified in *LPL* (four cases, 66.7%), followed by *APOA5* (one case, 16.7%) and *LMF1* (one case, 16.7%). One patient (14.3%) was a double heterozygote, carrying a pathogenic frameshift variant in *LPL* and a likely pathogenic missense variant in *GPIHBP1*. Overall, *LPL* was the most frequently involved gene, with variants identified in five FCS cases (71.4%) (Figure 2). Loss-of-function and missense variants were equally frequent in FCS (five variants each). Loss-of-function variants included frameshift variants (p.Lys40AsnfsTer4 and p.Leu392Ter in *LPL)*, a nonsense variant (p.Gln97Ter in *APOA5*), a splice-site variant (c.1019-3C > A in *LPL*), and a large-scale duplication in *LPL*, and were predominantly identified in *LPL* (80.0%) (Supplementary Table S1).

Among variant-positive MCS patients, seven (30.4%) carried a single pathogenic or likely pathogenic variant in one of the canonical chylomicronemia genes in the heterozygous state. Three patients (13.0%) carried a pathogenic or likely pathogenic variant together with an additional VUS, including one case of compound heterozygosity and two cases of double heterozygosity. Similar to FCS, most patients carried pathogenic or likely pathogenic variants in *LPL* (70.0%). In addition, 13 patients (56.5%) carried only VUSs in either a heterozygous (12 cases, 52.2%) or compound heterozygous (one case, 4.3%) state (Figure 2). Overall, 20 variants were observed in variant-positive MCS cases, with missense variants predominating (18 variants, 90.0%). Loss-of-function variants included two frameshift variants (p.Tyr194GlyfsTer69 in *APOA5* and p.Leu37SerfsTer4 in *APOC2*). The majority of identified variants were VUSs (12 variants, 60.0%) (Supplementary Table S1).

#### 2.2.3. *APOE* Variants and the ε2/ε2 Genotype

Rare *APOE* variants or the ε2/ε2 genotype were identified in 23 patients with severe HTG (18.7%). Eleven patients (8.9%) carried the ε2/ε2 genotype associated with the autosomal-recessive form of FD, while rare *APOE* variants linked to autosomal-dominant FD were detected in 12 patients (9.8%). All rare *APOE* variants were missense, and the most frequent was the likely pathogenic p.Arg154Cys variant (41.7% of cases). Among carriers of rare *APOE* variants, the ε2/ε1 genotype was observed in 25.0% of cases (Figure 1, Table A2).

### 2.3. Accumulation of Common TG-Raising Variants

PRS was calculated for all 123 participants to assess the contribution of common TG-raising variants to severe HTG. An extreme polygenic burden was defined as a PRS > 90th percentile based on the score distribution in the population-based sample (Section 4.4).

**Figure 3:**
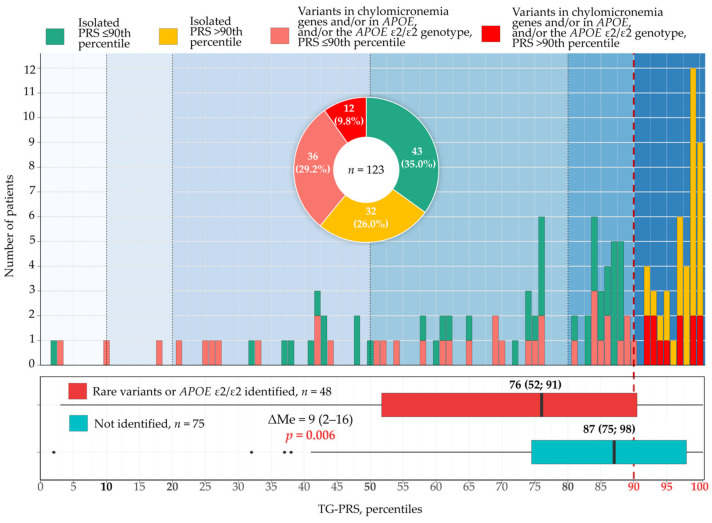
Distribution of 123 patients with severe HTG according to PRS percentiles and genetic status. The donut chart illustrates the spectrum of identified genetic determinants of HTG. Stacked bar plots show the distribution of patients across PRS percentiles (0–100). Bar height represents the total number of patients per percentile, while colored segments indicate the number of patients within each category. Blue background shading, increasing in intensity, indicates PRS percentile groups: <10, 10–19, 20–49, 50–79, 80–89, and 90–100. Horizontal boxplots summarize PRS percentile distributions according to rare genetic determinants of HTG: central lines indicate medians, box limits represent upper and lower quartiles, and horizontal lines represent 1.5 times the quartile range. Median (25th; 75th percentiles) is labeled above each boxplot. Group differences were assessed using the Mann–Whitney U test; ΔMe and 95% CI were estimated via the Hodges–Lehmann method. ΔMe—median difference; PRS—polygenic risk score; TG—triglycerides.

Overall, 44 patients (35.8%) had an extreme polygenic contribution to TG levels. Among carriers of rare variants in chylomicronemia genes and/or in *APOE*, including the ε2/ε2 genotype, 12 of 48 individuals (25.0%), corresponding to 9.8% of all HTG cases, also had PRS > 90th percentile, indicating a combined genetic etiology of severe HTG and a potentially more severe phenotype. In contrast, among those without these genetic determinants, 32 of 75 (42.7%), representing 26.0% of all HTG cases, had PRS > 90th percentile, suggesting an isolated extreme polygenic contribution (Figure 3).

Among the remaining 43 cases (35.0%) without rare variants, the ε2/ε2 genotype, or an extreme PRS, 33 individuals had PRS in the 50–90th percentile range, and 10 had PRS < 50th percentile (Figure 3).

In the study cohort, the median PRS percentile was 85 (62; 97). PRS percentiles were higher in patients without rare variants or the *APOE* ε2/ε2 genotype than in those with these genetic determinants: 87 (75; 98) vs. 76 (52; 91), respectively (ΔMe 9, 95% confidence interval (CI): 2–16; *p* = 0.006) (Figure 3).

### 2.4. Overlap Between Chylomicronemia Gene Variants, APOE, and an Extreme PRS

**Figure 4:**
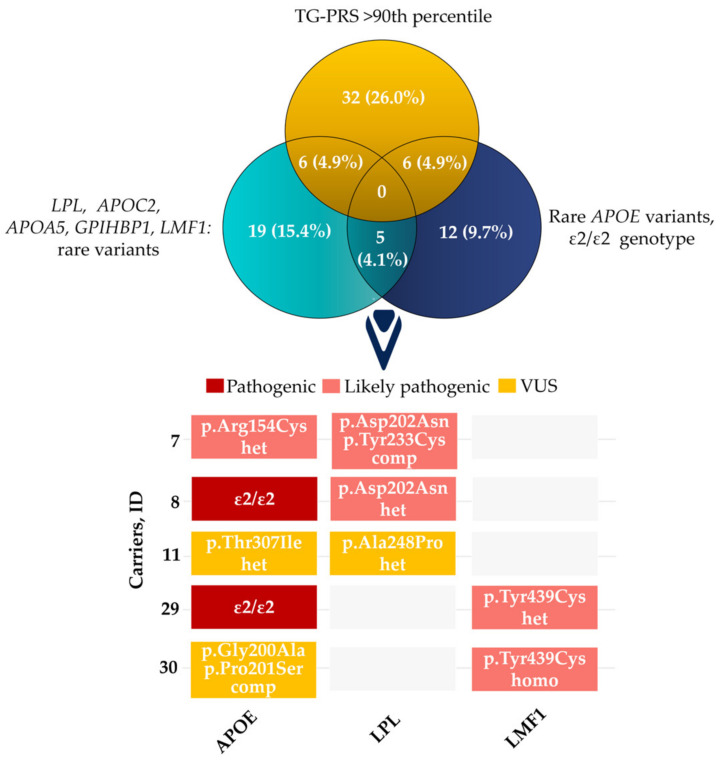
Overlap between chylomicronemia gene variants, *APOE*, and an extreme PRS. The Venn diagram shows the number of patients carrying variants in chylomicronemia genes, *APOE*, or both. PRS > 90th percentile, isolated or in combination with rare chylomicronemia variants or *APOE* (including ε2/ε2), are also indicated. The patient–gene matrix details the genetic spectrum of the five overlap cases, including involved genes, specific variants, zygosity, and variant classification.

Overall, major genetic determinants of severe HTG were verified in 80 individuals (65.0%). Overlap between rare variants in chylomicronemia genes and *APOE* was identified in five patients (4.1%). One patient was compound heterozygous for likely pathogenic variants in *LPL* and also carried a likely pathogenic *APOE* variant associated with FD. Another was homozygous for a likely pathogenic *LMF1* variant and additionally carried two VUSs in *APOE*. A third patient carried VUSs in both *LPL* and *APOE*, and the remaining two patients had the ε2/ε2 genotype and also carried likely pathogenic variants in *LPL* or *LMF1*, respectively (Figure 4). The median PRS percentile was 54 (42; 69) and none of these patients had an extreme PRS.

### 2.5. Comparison of Clinical Characteristics and PRS Percentile Across Genetically Defined Groups

**Figure 5:**
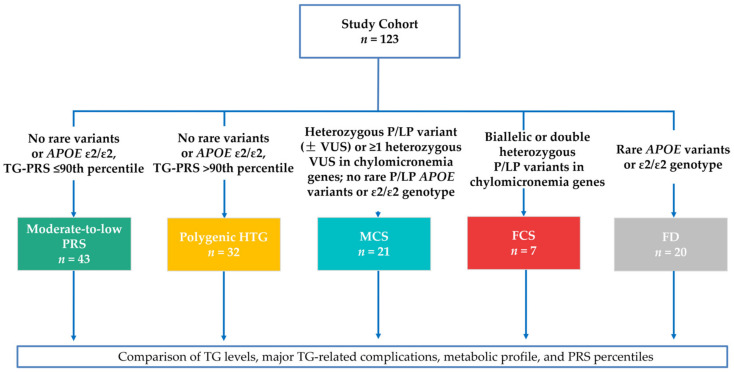
Genetic groups for comparison of clinical characteristics and TG-PRS. FCS—familial chylomicronemia syndrome; FD—familial dysbetalipoproteinemia; HTG—hypertriglyceridemia; LP—likely pathogenic; MCS—multifactorial familial chylomicronemia syndrome; P—pathogenic; PRS—polygenic risk score; TG—triglycerides; VUS—variant of uncertain significance.

We further categorized the cohort into five groups according to genetic findings (Figure 5). In cases with overlapping *APOE*-related findings and rare variants in chylomicronemia genes (Figure 4), group assignment was based on the genetic determinant with the strongest predicted causal effect (patients 7 and 30 were assigned to the FCS group, patients 8 and 29 to the FD group, and patient 11 to the MCS group).

We then assessed differences in TG levels, major TG-related complications, metabolic profile, and PRS percentiles across genetically defined groups (moderate-to-low PRS, polygenic HTG, MCS, FCS, and FD).

The clinical and demographic characteristics of the five genetic groups are presented in Table 2. Supplementary Table S1 provides the clinical and genetic characteristics for each individual with the identified rare genetic determinants of severe HTG.

After adjustment for sex and age, BMI was lower in FCS than in the FD, polygenic HTG, and moderate-to-low PRS groups, with mean differences of −5.6 kg/m^2^ (95% CI: −10.0 to −1.3; *p* = 0.012), −5.7 kg/m^2^ (95% CI: −9.9 to −1.5; *p* = 0.008), and −6.8 kg/m^2^ (95% CI: −11.1 to −2.6; *p* = 0.002), respectively. BMI was higher in the moderate-to-low PRS group than in MCS by 3.0 kg/m^2^ (95% CI: 0.1−5.9; *p* = 0.040). No significant differences were observed across the remaining groups (Supplementary Table S2).

**Figure 6:**
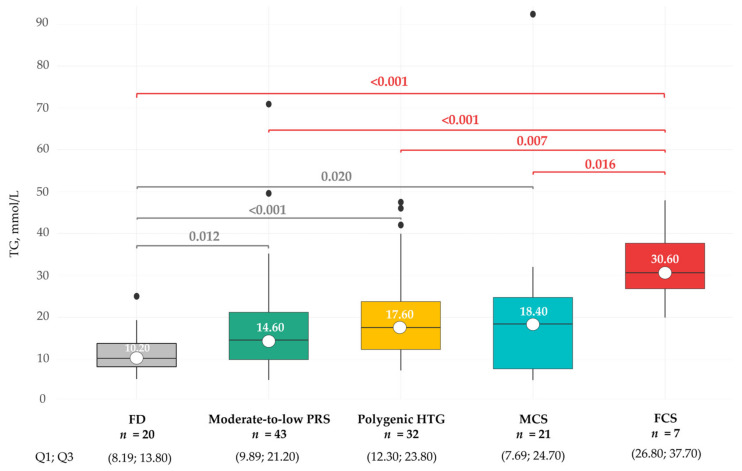
Distribution of TG levels across genetic groups. Boxplots show medians (white dots), first and third quartiles (lower and upper box limits), and whiskers represent 1.5 times the quartile range. Black dots represent outliers. Group comparisons were performed using a robust linear model on log_2_-transformed TG levels, adjusted for sex, age, and LLT. Pairwise comparisons between groups were based on estimated marginal means and are presented as back-transformed ratios. Only significant differences (*p* < 0.05) are indicated. Statistical significance annotations (connecting brackets and *p*-values) are color-coded according to the corresponding group. *p*-values are not corrected for multiple comparisons. Holm–Bonferroni-adjusted *p*-values are provided in Supplementary Table S2. FCS—familial chylomicronemia syndrome; FD—familial dysbetalipoproteinemia; HTG—hypertriglyceridemia; MCS—multifactorial familial chylomicronemia syndrome; PRS—polygenic risk score; TG—triglycerides.

After adjustment for sex, age, and lipid-lowering therapy (LLT), TG levels were significantly higher in FCS than in all other genetic groups. In contrast, TG levels were significantly lower in FD than in the other groups. TG levels were comparable across polygenic HTG, MCS, and moderate-to-low PRS groups (Figure 6, Supplementary Table S2).

**Figure 7:**
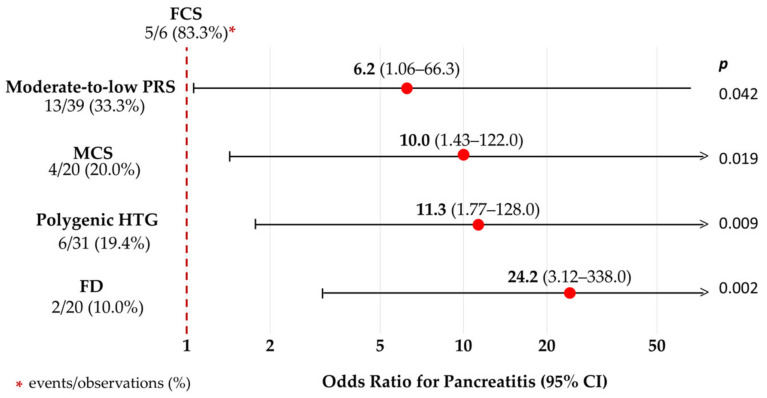
Pancreatitis risk: FCS vs. other genetic HTG groups. FCS was used as the index group for interpretation. The vertical dashed line indicates the null effect (odds ratio = 1). Odds ratios (red dots) and 95% CI were estimated using Firth penalized logistic regression adjusted for sex and age. Pairwise comparisons were obtained within the same model framework by varying the reference category. *p*-values are based on Firth profile likelihood tests from the covariate-adjusted model. *p*-values are unadjusted for multiple comparisons. Holm–Bonferroni-adjusted *p*-values are provided in Supplementary Table S2. CI—confidence interval; FCS—familial chylomicronemia syndrome; FD—familial dysbetalipoproteinemia; HTG—hypertriglyceridemia; MCS—multifactorial familial chylomicronemia syndrome; PRS—polygenic risk score.

The prevalence of pancreatitis differed across five genetic groups (*p* = 0.008) (Table 2). In a logistic regression adjusted for sex and age, FCS was associated with higher odds of pancreatitis compared with the moderate-to-low PRS, MCS, polygenic HTG, and FD groups (Figure 7). The wide CIs reflect limited precision due to the small sample size of this group, which is expected given the rare prevalence of the disease. The risk of pancreatitis was comparable across the FD, polygenic HTG, MCS, and moderate-to-low PRS groups (Supplementary Table S2).

**Figure 8:**
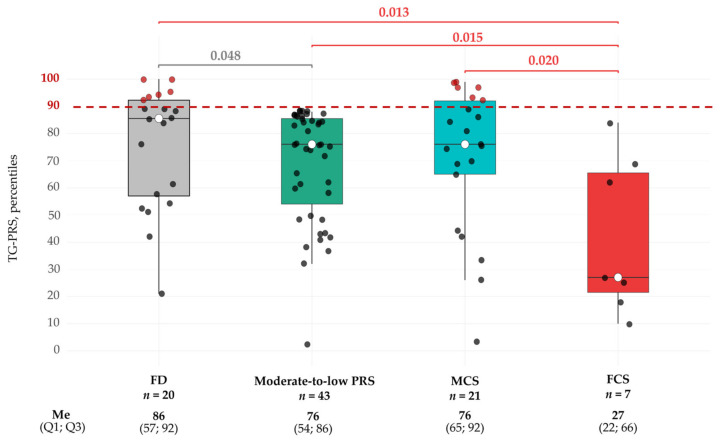
Distribution of PRS percentiles across genetic groups. Boxplots show medians (white dots), first and third quartiles (lower and upper box limits), and whiskers represent 1.5 times the quartile range. Individual PRS values are shown as black dots, patients with PRS > 90th percentile (red dash line) are highlighted in red. Pairwise comparisons were performed using the Mann–Whitney U test. Only significant differences (*p* < 0.05) are indicated. Statistical significance annotations (connecting brackets and *p*-values) are color-coded according to the corresponding group. *p*-values are unadjusted for multiple comparisons. Holm–Bonferroni-adjusted *p*-values are provided in Supplementary Table S2. FCS—familial chylomicronemia syndrome; FD—familial dysbetalipoproteinemia; MCS—multifactorial familial chylomicronemia syndrome; Me—median; PRS—polygenic risk score; TG—triglycerides.

In pairwise comparisons, PRS percentiles were lower in the FCS than in the MCS (ΔMe −30, 95% CI: −62 to −6; *p* = 0.020), moderate-to-low PRS (ΔMe −25, 95% CI: −58 to −4; *p* = 0.015), and FD groups (ΔMe −32, 95% CI: −66 to −10; *p* = 0.013). No extremely elevated PRS were observed in FCS. PRS percentiles were higher in FD than in the moderate-to-low PRS group (ΔMe 8, 95% CI: 0−15; *p* = 0.048), with borderline significance. The PRS distribution was comparable between FD and MCS and between MCS and the moderate-to-low PRS group (Figure 8, Supplementary Table S2).

## 3. Discussion

This study analyzed the genetic determinants of severe HTG. The main strength is the integrative genetic approach, which combined analysis of the canonical chylomicronemia genes, *APOE* variants, the ε2/ε2 genotype, and TG-PRS. This approach also allowed us to compare the clinical characteristics and PRS distribution across the moderate-to-low PRS, polygenic HTG, MCS, FCS, and FD groups. This is the first study providing a comprehensive assessment of the genetic determinants of severe HTG in an Eastern European cohort.

### 3.1. Genetic Determinants of Severe HTG

Major genetic determinants of severe HTG were identified in 65.0% of individuals. This yield is broadly consistent with previous reports (24.3–67.5%) [25,26,27,33,34,35,36]. Direct comparisons, however, are limited by differences in TG thresholds, the spectrum of genes analyzed, variant classification methods, PRS assessment, and population ancestry, which may influence the spectrum and frequency of detected variants [33].

Our results further underscore the genetic heterogeneity of severe HTG: 25.1% of individuals carried rare variants in canonical chylomicronemia genes or *APOE*, including the ε2/ε2 genotype; 4.1% had overlap between these findings; 9.8% had both rare variants or the ε2/ε2 genotype and an extreme PRS; and 26.0% had isolated polygenic HTG. The remaining 35.0% without major genetic determinants had moderate-to-low PRS.

#### 3.1.1. Chylomicronemia Genes

The prevalence of rare-variant carriers across five canonical chylomicronemia genes was 24.4%, consistent with rates reported in large international cohorts, including 23.6% and 24.3% in Canadian and UK studies of extreme HTG [27,34], and similar to the 15.5% observed in a combined Canadian and US cohort [25]. The higher prevalence in the Italian LIPIGEN cohort (63.9%) likely reflects differences in study design, including clinically confirmed FCS, as well as analysis of additional genes (*CREB3L3* and *GPD1*) [35].

At the genotype level, single heterozygotes predominated (63.3% of variant carriers), while biallelic or double heterozygotes were less common (36.6%). Accordingly, genetically confirmed FCS was relatively infrequent in our cohort (5.7%) compared with international reports (12.9–48.0%) [33,34,35]. The only previous Russian study reported a higher prevalence of biallelic variants in canonical genes (14.6%). However, it focused on patients with extreme HTG (median TG 39.0 mmol/L) and clinically confirmed FCS, which likely explains the differences [37]. Emerging evidence supporting the pathogenicity of identified VUSs may increase the prevalence of genetically defined FCS in our cohort to 8.9%.

Rare variants were predominantly identified in *LPL* (53.6%), accounting for the majority of causative variants across chylomicronemia genes. Consistently, *LPL* variants were present in 71.4% of FCS cases, confirming their central role in the genetic architecture of FCS. This predominance has been previously observed in European individuals from the UK cohort [33] and is consistent with the LIPIGEN study, where *LPL* variants were detected in 72.0% of FCS cases [35].

Although missense variants predominated in the overall spectrum of chylomicronemia genes, we identified seven loss-of-function variants, 57.1% of which were located in *LPL*. Furthermore, in the genetic architecture of FCS, loss-of-function variants were also predominantly observed in *LPL*. LPL is the key catalytic enzyme that hydrolyzes TG in TG-rich lipoproteins. Loss-of-function variants typically result in a marked reduction or complete loss of LPL activity, particularly in biallelic carriers. As a result, affected patients may present with a more severe phenotype and a reduced response to standard TG-lowering therapy that depend on LPL activity. In contrast, missense variants may be associated with variable residual LPL activity depending on their functional impact [3,12].

We also identified four novel pathogenic variants and six missense VUSs in chylomicronemia genes. The identification of novel variants expands the known spectrum of HTG-associated variants and improves variant interpretation and genetic diagnosis. As additional evidence accumulates, including segregation with the phenotype in affected family members or unrelated patients, as well as results from functional studies, VUSs may be reclassified as pathogenic variants. Such reclassification would improve the genetic diagnosis of severe HTG, facilitate cascade screening, and contribute to more accurate risk stratification and therapeutic decision-making.

#### 3.1.2. *APOE* Gene

We extended the analysis beyond the canonical chylomicronemia genes to include rare *APOE* variants and the ε2/ε2 genotype, both associated with FD. *APOE* encodes apolipoprotein E, a key ligand mediating receptor-dependent hepatic clearance of cholesterol-enriched remnants. Interactions between FD-associated genetic variants and additional modifying factors (metabolic or genetic) promote the accumulation of remnant lipoproteins and the development of the FD phenotype [14,16,29].

This approach identified additional genetic determinants in 18 individuals (+14.6% relative to carriers of variants in chylomicronemia genes) and detected overlap between chylomicronemia variants and *APOE* in five patients. Overall, rare *APOE* variants or the ε2/ε2 genotype were present in 18.7% of the cohort, representing the significant component of the genetic architecture of severe HTG, comparable to rare variants in chylomicronemia genes.

This is the first study to evaluate both the ε2/ε2 genotype, associated with autosomal-recessive FD, and rare *APOE* variants linked to autosomal-dominant FD alongside the canonical chylomicronemia genes. The prevalence of the ε2/ε2 genotype in our cohort (8.9%) was higher than that reported in a Turkish cohort with severe HTG (1.5%) [36], which may reflect population-specific differences [23].

#### 3.1.3. Polygenic HTG

Polygenic predisposition to elevated TG levels can be calculated using a TG-PRS, which reflects the cumulative burden of multiple common TG-raising variants across genes involved in TG-rich lipoprotein metabolism. The accumulation of such variants has an additive effect on TG levels and may contribute to a severe HTG phenotype, even in the absence of rare TG-associated variants [25,26]. In this study, we used a TG-PRS based on 40 TG-raising variants.

An extreme polygenic contribution to TG levels was present in 35.8% of individuals, representing the most common genetic component of severe HTG in this cohort. Isolated polygenic HTG was observed in 26.0% of individuals, suggesting that a predominant polygenic burden may contribute to a severe HTG phenotype, potentially in interaction with non-genetic factors. In the Dron 2019 and Dron 2020 studies, isolated polygenic HTG was the most common type of genetically defined severe HTG (32.0% and 26.4%, respectively) [25,27]. Our results demonstrate that severe HTG may also be caused by *APOE* variants. Together with rare chylomicronemia gene variants, they accounted for 39.0% of cases, a prevalence comparable to that of an extreme PRS.

The overlap of extreme PRS with rare variants, including the ε2/ε2 genotype (9.8% of all patients), further extends the complexity of the genetic architecture of severe HTG. These findings are similar to the Dron 2020 study, in which 6.3% of patients had both chylomicronemia gene variants and an extreme PRS [27].

#### 3.1.4. Moderate-to-Low PRS Cases

In the remaining 35.0% of patients, no major genetic determinants of severe HTG were identified. These individuals nonetheless had a relatively high PRS distribution (median 76th percentile), which may have contributed to elevated TG levels. Among these cases, 76.7% had PRS in the 50–90th percentile range, reflecting a moderate PRS, while 23.3% had low PRS (<50th percentile). Additional drivers of severe HTG in these patients may include rare variants in noncanonical HTG genes that were not evaluated in this study, such as *GPD1* and *CREB3L3*. In particular, the inclusion of *CREB3L3* has been shown to increase the genetic diagnostic yield of severe HTG and is likely more prevalent in patients with MCS [35,37]; genes associated with secondary causes of HTG, such as monogenic diabetes or inherited forms of lipodystrophy; and non-genetic factors. Although lifestyle-related factors, including dietary habits, were not evaluated in this study, we observed a relatively high prevalence of metabolic syndrome components (40.0%) within this group, suggesting a potential additive effect of these factors with PRS on extremely elevated TG levels.

### 3.2. Differences in Main Clinical Characteristics and PRS Distribution

Clinical differences among FCS, MCS, and the ”rare-variant-negative” groups have been well-described [33,35,38,39], but most previous studies did not assess PRS or include FD for comparison.

In our cohort, BMI was significantly lower in FCS than in the moderate-to-low PRS, polygenic HTG, and FD groups, underlining a lower metabolic burden in FCS. These findings are consistent with LIPIGEN [35] and the Paquette 2021 [38] studies. However, these studies did not assess TG-PRS, and their “rare-variant-negative” group may have included patients with polygenic HTG. Notably, BMI was comparable between FCS and MCS, likely reflecting the overall high metabolic burden across cohorts and overlapping BMI distribution.

All genetic groups had extreme TG levels (>10.0 mmol/L), but FCS demonstrated the highest values, similar to previous chylomicronemia studies [35,38,39]. In contrast, TG levels were significantly lower in FD than in the other groups, a result we report here for the first time, which likely reflects differences in TG metabolism.

The prevalence of pancreatitis in FCS was 83.3%, with 6-to-11-fold higher odds than in the moderate-to-low PRS, MCS, and polygenic HTG groups, consistent with previous reports [33,35,38]. Although, in contrast to our findings, which may reflect comparable PRS and TG burden between moderate-to-low PRS and MCS, the Paquette 2021 study reported a significant difference between MCS and “rare-variant-negative” groups [38]. In addition, the odds of pancreatitis in our cohort were up to 24-fold higher in FCS than in FD, consistent with the lower TG levels observed in FD.

In addition, we compared PRS percentiles across the FCS, MCS, moderate-to-low PRS, and FD groups. Lower PRS percentiles in FCS and the absence of extremely elevated PRS align with FCS’s predominantly monogenic architecture. Differences between FD and the moderate-to-low PRS group were borderline.

We did not observe differences in CAD prevalence across five groups, consistent with previous studies demonstrating comparable rates between FCS and MCS in LIPIGEN [35] and in a UK cohort, where borderline differences became nonsignificant after adjustment for age, sex, and BMI [33].

Despite distinct genetic determinants across FCS, MCS, polygenic HTG, and moderate-to-low PRS groups, their clinical comparisons show that patients with FCS are likely to benefit the most from genetic testing, which is consistent with the Spagnuolo 2025 study [39]. The contribution of an elevated TG-PRS toward phenotype severity requires further analysis [40]. A previous study has shown that individuals carrying both rare pathogenic variants in chylomicronemia genes and a high TG-PRS have more severe HTG and a higher risk of acute pancreatitis than those without such variants and with low PRS [41]. We were unable to replicate this comparison in our cohort due to the limited number of MCS individuals with PRS > 90th percentile. In FD patients, the phenotype may develop at lower TG levels (≥1.5 mmol/L). Identification of *APOE* variants or the ε2/ε2 genotype, as well as assessment of TG-PRS, may improve early detection and risk stratification in FD, as previously reported [29].

## 4. Materials and Methods

### 4.1. Study Subjects

The study sample was derived from the genotyped subset of the Biobank of the National Medical Research Center (NMRC) for Therapy and Preventive Medicine (Moscow, Russia) [28]. In the present analysis, we included unrelated index patients aged ≥ 18 years with fasting TG levels > 5.0 mmol/L *(n* = 123). These patients were recruited within an ongoing research project focused on chronic noncommunicable diseases, including lipid metabolism disorders [42].

Although a TG level > 5.0 mmol/L corresponds to moderate HTG according to the 2021 European Atherosclerosis Society consensus [2], the cutoff was selected to reduce underdiagnosis of severe cases (>500 mg/dL or >5.65 mmol/L [2]) due to TG variability and the use of LLT. Only five individuals (4.1%) had TG levels between 5.0 and 5.65 mmol/L, indicating minimal inclusion of milder cases. This approach allowed capturing a broader spectrum of genetically determined HTG.

### 4.2. Clinical and Biochemical Data

The retrospective clinical and biochemical data included age, sex, BMI, smoking status, and the presence of hypertension, glucose metabolism disorders, hypothyroidism, pancreatitis, and CAD, including myocardial infarction and coronary revascularization. The presence of CAD was based on medical records and was diagnosed according to current European clinical guidelines. Early-onset CAD was defined as age at diagnosis <55 years in men and <60 years in women. Glucose metabolism disorders were defined as clinical diagnoses of either type 2 diabetes, impaired glucose tolerance, or impaired fasting glucose. Metabolic syndrome components included at least two of the following: hypertension, BMI ≥ 30.0 kg/m^2^, or glucose metabolism disorders. The presence of xanthomas was considered when these data were available. The type of LLT was also analyzed.

TG levels were reported in mmol/L and were measured using the Abbott Architect C-8000 system (Abbott Laboratories, North Chicago, IL, USA). To capture maximal phenotypic severity, the highest documented fasting TG value from the medical record was used for each participant. For the 29 patients receiving LLT, no TG recalculation was performed.

### 4.3. Genetic Analysis

#### 4.3.1. NGS

Blood samples were stored at −32 °C in the Biobank of the NMRC for Therapy and Preventive Medicine [42]. NGS was performed using custom target panel (*n* = 123) designs with the NextSeq 550 (Illumina, San Diego, CA, USA) platform, as previously described [13]. Five canonical chylomicronemia genes—*LPL* (HGNC:6677), *APOC2* (HGNC:609), *APOA5* (HGNC:17288), *GPIHBP1* (HGNC:24945), and *LMF1* (HGNC:14154)—as well as FD gene *APOE* (HGNC:613) were analyzed. Sanger sequencing was performed using the Applied Biosystem 3500 Genetic Analyzer (Thermo Fisher Scientific, Waltham, MA, USA) following the manufacturer’s protocol.

#### 4.3.2. Bioinformatic Analysis and Clinical Interpretation

All steps of the bioinformatic analysis have been described previously [13]. Briefly, the GRCh38/hg38 reference genome was selected for aligning paired-end reads. The generated VCF files contained a list of variants, their genomic coordinates, coverage data, and other characteristics. For the current study, principal component analysis (PCA) was performed on individual genotypes using Hail v.0.2.130 [43]. For this analysis, variants with a minor allele frequency < 5.0% were excluded, and linkage disequilibrium pruning was applied (r^2^ = 0.2). Samples were projected onto reference populations from the 1000 Genomes Project Phase 3, and all analyzed individuals clustered within the European population. No outliers were detected or removed.

The following canonical transcripts were used for variant annotation: NM_000237.3 (*LPL*), NM_000483.5 (*APOC2*), NM_052968.5 (*APOA5*), NM_178172.6 (*GPIHBP1*), NM_022773.4 (*LMF1*), and NM_000041.4 (*APOE*). Variants with a minor allele frequency <0.5% across populations in the Genome Aggregation Database v4.1.0 [44] and predicted to have moderate or high impact were selected for evaluation according to the ACMG/AMP2015 guidelines [45]. For *APOE*, the ε2/ε2 genotype was additionally considered and identified as described previously [23]. The following types of variants are reported in the article: pathogenic, likely pathogenic, or VUS.

Combined Annotation Dependent Depletion (CADD v1.7 for GRCh38) was used to predict the impact of single-nucleotide variants as well as deletion variants [46]. Scaled CADD scores (PHRED-like scaled C-scores) were obtained to assess the deleteriousness of identified variants [47]. Variants with a PHRED score ≥ 20 were considered potentially pathogenic. In this case, the criterion PP3 ACMG/AMP2015 was used. For splice variants we used the SpliceAI Δ score obtained from CADD v.1.7 [47].

We reported variants as novel when they had not been previously described in public databases or in the literature, including our own previous studies.

A genetic diagnosis of FCS was established based on one of the following criteria: (1) homozygous or compound heterozygous pathogenic or likely pathogenic variants in one of the five canonical chylomicronemia genes (*LPL*, *APOC2*, *APOA5*, *GPIHBP1*, *LMF1*); or (2) pathogenic or likely pathogenic variants in two of these genes (double heterozygotes).

Variant-positive MCS was defined by the presence of the following types of variants in chylomicronemia genes: (1) a heterozygous pathogenic or likely pathogenic variant; or (2) a heterozygous pathogenic or likely pathogenic variant with an additional VUS; or (3) one or more heterozygous VUS [3].

The FD genetic group was defined by the presence of the ε2/ε2 *APOE* genotype or rare *APOE* variants [13].

For comparison of clinical and genetic characteristics, cases with overlapping *APOE*-related findings and rare variants in chylomicronemia genes were assigned into groups based on the genetic determinant with the strongest predicted causal effect.

### 4.4. Polygenic Risk Score for Elevated TG Levels

PRS was calculated for all study participants using the β-coefficients from the original study [48], which previously demonstrated significant associations with TG levels (40 variants, Supplementary Table S3) in the population from the European part of Russia [49]. In the present study, the PRS percentile distribution was acquired from this joint population sample (*n* = 2490) and was used to calculate participants’ PRS percentiles. Polygenic HTG was defined as a weighted PRS > 90th percentile [49].

### 4.5. Ethical Statement

The study was conducted in accordance with the Declaration of Helsinki and the National Standard of the Russian Federation “Good Clinical Practice (GCP)” (GOST R 52379-2005 [50]) and was approved by the Independent Ethics Committee of the NMRC for Therapy and Preventive Medicine (protocols number 05-05/15, dated 9 June 2015, and number 07-05/20, dated 26 November 2020). To comply with these regulations, as well as Article 93 of the Federal Law “On the Fundamentals of Health Protection of Citizens of the Russian Federation” (dated 21 November 2011, No. 323-FZ), each participant provided written informed consent for the processing of their personal data. The database containing clinical, biochemical, and genetic data was deidentified and encrypted to ensure participant confidentiality.

### 4.6. Statistical Analysis

Statistical analyses were performed using R version 4.4.3 (R Foundation for Statistical Computing, Vienna, Austria) [51].

Continuous variables (age, BMI, PRS percentiles, and TG levels) were summarized as Me (25th; 75th percentiles) to provide distribution-robust descriptive statistics that are not sensitive to limited and heterogeneous subgroup sizes. Categorical variables were presented as absolute numbers and percentages.

For comparisons of continuous variables between two independent groups, the Mann–Whitney U test was used. In these cases, the pseudomedian difference was estimated using the Hodges–Lehmann method with a corresponding 95% CI. For comparisons across five genetic groups, the Kruskal–Wallis test was used for continuous variables, and the two-sided Fisher’s exact test was applied for categorical variables.

TG levels were log_2_-transformed prior to analysis. Between-group differences in TG levels were assessed using multivariable linear regression models adjusted for sex, age, and LLT. For BMI, models were adjusted for sex and age. Robust standard errors (HC3) were applied. Estimated marginal means derived from these models were used for pairwise comparisons between genetic groups. For TG levels, the results are presented as fold-changes (2^estimate, i.e., back-transformed from the log_2_ scale), and for BMI as mean differences (kg/m^2^), both with 95% CI and *p*-values.

Firth penalized logistic regression (logistf package version 1.26.1 [52]) was used to evaluate the association between genetically defined groups and the risk of pancreatitis, adjusted for sex and age. Pairwise comparisons were obtained within the same model framework by varying the reference category. Results are reported as odds ratios with 95% CI and corresponding *p*-values.

*p*-values reported in the main analyses are unadjusted for multiple comparisons, as comparisons were prespecified and performed between biologically defined genetic groups. Holm–Bonferroni-adjusted *p*-values are provided in Supplementary Table S2. *p*-values of less than 0.05 were considered statistically significant.

Data visualization was performed using the ggplot2 version 4.0.0 [53], patchwork version 1.3.2 [54], and VennDiagram version 1.8.2 [55].

### 4.7. Limitations

This study has several limitations. The genetic analysis did not include noncanonical chylomicronemia genes or genes associated with secondary causes of HTG, which may have increased the diagnostic yield of genetically defined cases. Several patients in the MCS group carried VUS in the compound heterozygous or double-heterozygous state. Future reclassification of these variants as pathogenic may increase the proportion of genetically defined FCS. However, given the limited number of such patients (*n* = 4), a significant impact on the observed genotype–phenotype associations in the current study is unlikely. Six patients in the MCS group and six in the FD group carried both rare pathogenic variants and high TG-PRS, suggesting a potentially more severe phenotype. However, their limited number prevented separate subgroup analyses. In addition, the cohort included only individuals of European ancestry, limiting generalizability to other populations. The clinical data were retrospectively derived from a project not specifically designed for patients with chylomicronemia, limiting the completeness of comparisons of related complications. In addition, lifestyle-related factors, such as dietary habits, were not available for evaluation. Finally, the relatively small sample size, particularly in the FCS group, may have reduced statistical power to detect less pronounced associations.

## 5. Conclusions

Severe HTG demonstrated a highly heterogeneous genetic architecture in this cohort. Analysis of canonical chylomicronemia genes identified 24.4% of cases. A broader genetic approach, including *APOE* variants and the ε2/ε2 genotype, detected an additional 14.6% of non-overlapping cases and overlap with chylomicronemia variants in 4.1%. Assessment of TG-PRS further identified 26.0% with isolated polygenic HTG and 9.8% with overlap, increasing the overall yield of major genetic determinants to 65.0%. The remaining 35.0% had moderate-to-low PRS. Among genetically defined HTG groups, FCS had the most distinct clinical profile. Further studies are required to determine whether elevated TG-PRS modifies phenotype severity in carriers of rare variants.

## Figures and Tables

**Table 1:** Clinical and demographic characteristics.

Parameter	Total Cohort (*n* = 123)	TG > 5.0 to 10.0 mmol/L (*n* = 34)	TG > 10.0 mmol/L (*n* = 89)
Men, *n* (%)	73 (59.3)	24 (70.6)	49 (55.1)
Age, years, Me (Q1; Q3)	48 (41; 54)	47 (37; 56)	49 (42; 53)
Smoking (current or former smokers), *n* (%)	59 (50.0) *n* = 118	19 (59.4) *n* = 32	40 (46.5) *n* = 86
Hypertension, *n* (%)	77 (63.1) *n* = 122	22 (66.7) *n* = 33	55 (61.8)
BMI, kg/m^2^, Me (Q1; Q3)	29.0 (26.4; 32.0) *n* = 121	29.6 (25.5; 34.6) *n* = 33	28.8 (26.6; 31.6) *n* = 88
Glucose metabolism disorders, *n* (%) ^1^	47 (38.2)	11 (32.4)	36 (40.4)
Metabolic syndrome components, *n* (%) ^2^	42 (35.0) *n* = 120	15 (46.9) *n* = 32	27 (30.7) *n* = 88
Cutaneous xanthomas, *n* (%)	21 (19.3) *n* = 109	4 (14.3) *n* = 28	17 (21.0) *n* = 81
Pancreatitis, *n* (%)	30 (25.9) *n* = 116	2 (6.5) *n* = 31	28 (32.9) *n* = 85
CAD, *n* (%)	29 (23.6)	12 (35.3)	17 (19.1)
Age at onset of CAD, years, Me (Q1; Q3)	48 (39; 54)	37 (36; 53)	48 (46; 54)
Maximal TG, mmol/L, Me (Q1; Q3) ^3^	14.80 (9.70; 23.10)	7.78 (6.03; 9.06)	19.80 (14.10; 26.30)
LLT, *n* (%) ^4^	29 (23.6)	12 (35.3)	17 (19.1)

^1^ Glucose metabolism disorders included type 2 diabetes, impaired glucose tolerance, and impaired fasting glucose. ^2^ Metabolic syndrome components included at least two of the following: hypertension, BMI ≥ 30.0 kg/m^2^, or glucose metabolism disorders. ^3^ Highest documented fasting TG value available in the medical record for each participant. ^4^ Any LLT at the time of inclusion. BMI—body mass index; CAD—coronary artery disease; LLT—lipid-lowering therapy; Me—median; TG—triglycerides.

**Table 2:** Clinical and demographic characteristics across five genetic groups.

Parameter	Moderate-to-Low PRS (*n* = 43)	Polygenic HTG (*n* = 32)	MCS (*n* = 21)	FCS (*n* = 7)	FD (*n* = 20)	*p*-Value ^1^
Men, *n* (%)	23 (53.5)	22 (68.8)	15 (71.4)	2 (28.6)	11 (55.0)	0.215
Age, years, Me (Q1; Q3)	49 (41; 54)	47 (41; 54)	48 (41; 55)	46 (37; 51)	48 (39; 52)	0.904
Smoking (current or former smokers), *n* (%)	18 (46.2) *n* = 39	19 (61.3) *n* = 31	13 (61.9)	1 (14.3)	8 (40.0)	0.124
Hypertension, *n* (%)	28 (66.7) *n* = 42	23 (71.9)	14 (66.7)	3 (42.9)	9 (45.0)	0.251
BMI, kg/m^2^, Me (Q1; Q3)	29.6 (27.8; 34.5)	29.2 (27.0; 32.2)	28.0 (25.0; 31.0)	23.0 (19.6; 27.2)	29.1 (26.6; 31.8)	0.022
Glucose metabolism disorders, *n* (%) ^2^	18 (41.9)	11 (34.4)	9 (42.9)	3 (42.9)	6 (30.0)	0.872
Metabolic syndrome components, *n* (%) ^3^	16 (40.0) *n* = 40	12 (37.5)	6 (28.6)	1 (14.3)	7 (35.0)	0.749
Cutaneous xanthomas, *n* (%)	10 (26.3) *n* = 38	4 (14.3) *n* = 28	2 (10.5) *n* = 19	0 *n* = 5	5 (26.3) *n* = 19	0.431
Pancreatitis, *n* (%)	13 (33.3) *n* = 39	6 (19.4) *n* = 31	4 (20.0) *n* = 20	5 (83.3) *n* = 6	2 (10.0)	0.008
CAD, *n* (%)	14 (32.6)	5 (15.6)	4 (19.0)	1 (14.3)	5 (25.0)	0.508
LLT, *n* (%) ^4^	10 (23.3)	4 (12.5)	7 (33.3)	3 (42.9)	5 (25.0)	0.270

^1^ *p*-values indicate differences between five groups. The Kruskal–Wallis test was used for continuous variables. For categorical variables, *p*-values were obtained using the two-sided Fisher’s exact test. ^2^ Glucose metabolism disorders included type 2 diabetes, impaired glucose tolerance, and impaired fasting glucose. ^3^ Metabolic syndrome components included at least two of the following: hypertension, BMI ≥ 30.0 kg/m^2^, or glucose metabolism disorders. ^4^ Any LLT at the time of inclusion. BMI—body mass index; CAD—coronary artery disease; FCS—familial chylomicronemia syndrome; FD—familial dysbetalipoproteinemia; HTG—hypertriglyceridemia; LLT—lipid-lowering therapy; MCS—multifactorial familial chylomicronemia syndrome; Me—median; PRS—polygenic risk score.

## Data Availability

The data used in this study, including individual genotype information, cannot be publicly disclosed according to the rules of the Ethics Committee of the National Medical Research Center for Therapy and Preventive Medicine. Deidentified data will be provided upon reasonable request by the corresponding author, Anastasia Blokhina (blokhina0310@gmail.com), or by the Ethics Committee of the National Medical Research Center for Therapy and Preventive Medicine (phone number +74995536810, secretarynec@gnicpm.ru). Proposals will be reviewed and approved by the investigators, local regulatory authorities, and the Ethics Committee of the National Medical Research Center for Therapy and Preventive Medicine. Once the proposal is approved, data can be transferred through a secure online platform after signing a data access agreement and a confidentiality agreement.

## Supplementary Materials

The following supporting information can be downloaded at https://www.mdpi.com/article/10.3390/ijms27125443/s1.

## Abbreviations

The following abbreviations are used in this manuscript:

BMI	Body mass index
CAD	Coronary artery disease
CADD	Combined annotation dependent depletion
CI	Confidence interval
FCS	Familial chylomicronemia syndrome
FD	Familial dysbetalipoproteinemia
HTG	Hypertriglyceridemia
LLT	Lipid-lowering therapy
MCS	Multifactorial chylomicronemia syndrome
NMRC	National Medical Research Center
PRS	Polygenic risk score
TG	Triglyceride
VUS	Variant of uncertain significance

## Appendix A

**Table A1:** Rare variants identified in chylomicronemia genes in the study cohort.

Gene, Total No. of Variants	Variant No.	Variant	Genomic Coordinates (GRCh38)	Reference Allele	Alternative Allele	Variant Type	HGVSc	HGVSp	Total AF gnomAD v4.1.0, %	ACMG Class	CADD PHRED v1.7	Carrier Count	Allele Count	Previously Reported
*LPL*, *n* = 15	1	Not registered	chr8:19948208	TA	T	Frameshift	c.120del	p.Lys40AsnfsTer4	Not reported	P	33.0	1	2	no
	2	rs375484335	chr8:19948266	G	A	Missense	c.175G>A	p.Gly59Arg	0.002106	VUS	28.2	1	1	yes
	3	Not registered	chr8:19951790	T	G	Missense	c.271T>G	p.Trp91Gly	Not reported	VUS	29.9	1	1	no
	4	rs118204069	chr8:19951856	T	C	Missense	c.337T>C	p.Trp113Arg	0.0006196	P	29.8	3	3	yes
	5	Not registered	chr8:19954138	C	G	Missense	c.560C>G	p.Pro187Arg	Not reported	VUS	28.5	1	1	no
	6	Not registered	chr8:19954182	G	A	Missense	c.604G>A	p.Asp202Asn	Not reported	LP	28.8	2	2	yes
	7	rs118204057	chr8:19954222	G	A	Missense	c.644G>A	p.Gly215Glu	0.03556	P	24.7	2	3	yes
	8	Not registered	chr8:19954276	A	G	Missense	c.698A>G	p.Tyr233Cys	Not reported	LP	28.1	1	1	yes
	9	Not registered	chr8:19954320	G	C	Missense	c.742G>C	p.Ala248Pro	Not reported	VUS	26.9	1	1	no
	10	rs118204080	chr8:19954333	T	C	Missense	c.755T>C	p.Ile252Thr	0.003779	P	25.3	1	1	yes
	11	rs773407951	chr8:19955883	A	G	Missense	c.818A>G	p.His273Arg	0.0006195	LP	26.1	1	1	yes
	12	Not registered	chr8:19959265	C	T	Missense	c.1024C>T	p.His342Tyr	Not reported	VUS	26.5	1	1	no
	13	Not registered	chr8:19960933	TC	T	Frameshift	c.1174del	p.Leu392Ter	Not reported	P	27.7	1	1	no
	14	rs2128839190	chr8:19959257	C	A	Splice-region variant	c.1019-3C>A	—	0.0001239	LP	SpliceAI Δ score = 0.67	1	1	yes
	15	Not registered	chr8:19955963 -19958022	—	—	2-kb duplication	c.897_1019-1238dup	—	Not reported	P	—	1	1	no
*APOA5*, *n* = 6	16	rs201079485	chr11:116790940	G	A	Nonsense	c.289C>T	p.Gln97Ter	0.01711	P	36.0	1	2	yes
	17	rs1940989106	chr11:116790636	AGGCTCTCGGCGTAT	A	Frameshift	c.579_592del	p.Tyr194GlyfsTer69	Not reported	P	33.0	1	1	yes
	18	rs2075291	chr11:116790676	C	A	Missense	c.553G>T	p.Gly185Cys	0.2916	VUS	18.79	6	6	yes
	19	Not registered	chr11:116790622	C	T	Missense	c.607G>A	p.Gly203Arg	Not reported	VUS	20.3	1	1	no
	20	Not registered	chr11:116790562	G	A	Missense	c.667C>T	p.Arg223Cys	Not reported	VUS	25.3	1	1	yes
	21	rs563462071	chr11:116790531	C	T	Missense	c.698G>A	p.Arg233Gln	0.002189	VUS	7.902	1	1	yes
*GPIHBP1*, *n* = 3	22	rs201685731	chr8:143215331	G	A	Missense	c.368G>A	p.Gly123Glu	0.04198	VUS	11.68	1	1	yes
	23	rs373297994	chr8:143215447	G	A	Missense	c.484G>A	p.Glu162Lys	0.005086	VUS	0.010	1	1	yes
	24	rs145844329	chr8:143215486	G	C	Missense	c.523G>C	p.Gly175Arg	0.07318	LP	6.949	1	1	yes
*APOC2*, *n* = 2	25	Not registered	chr19:44948752	TC	T	Frameshift	c.109del	p.Leu37SerfsTer4	Not reported	P	—	1	1	no
	26	rs120074114	chr19:44948767	A	C	Missense	c.122A>C	p.Lys41Thr	0.08605	VUS	17.25	1	1	yes
*LMF1*, *n* = 2	27	Not registered	chr16:954417	A	T	Missense	c.443T>A	p.Met148Lys	Not reported	VUS	23.2	1	1	no
	28	rs764885027	chr16:869983	T	C	Missense	c.1316A>G	p.Tyr439Cys	0.004649	LP	23.1	2	3	yes

ACMG—American College of Medical Genetics and Genomics/Association for Molecular Pathology; AF—allele frequency; CADD—combined annotation dependent depletion; del—deletion; dup—duplication; fs—frameshift; gnomAD—Genome Aggregation Database; HGVSc—Human Genome Variation Society coding sequence name; HGVSp—Human Genome Variation Society protein; LP—likely pathogenic; P—pathogenic; ter—termination codon; VUS—variant of uncertain significance.

**Table A2:** Rare *APOE* variants identified in the study cohort.

Gene, Total No. of Variants	Variant No.	Variant	Genomic Coordinates (GRCh38)	Reference Allele	Alternative Allele	Variant Type	HGVSc	HGVSp	Total AF gnomAD v4.1.0, %	ACMG Class	CADD PHRED v1.7	Carrier Count	*APOE* Genotype	Previously Reported
Rare *APOE* variants, *n* = 6	1	rs267606664	chr19:44908730	G	A	Missense	c.434G>A	p.Gly145Asp	0.01673	LP	23.2	3	ε2/ε1	yes
	2	rs121918393	chr19:44908756	C	T	Missense	c.460C>T	p.Arg154Cys	0.005642	LP	27.5	5	ε3/ε3	yes
	3	Not registered	chr19:44908895	G	C	Missense	c.599G>C	p.Gly200Ala	Not reported	VUS	5.685	1	ε3/ε3	yes
	4	Not registered	chr19:44908897	C	T	Missense	c.601C>T	p.Pro201Ser	Not reported	VUS	16.47	2	ε3/ε3 ε4/ε4	yes
	5	rs567353589	chr19:44908984	G	A	Missense	c.688G>A	p.Glu230Lys	0.002415	LP	18.30	1	ε3/ε3	yes
	6	rs770562611	chr19:44909216	C	T	Missense	c.920C>T	p.Thr307Ile	0.0002505	VUS	10.72	1	ε3/ε3	yes

ACMG—American College of Medical Genetics and Genomics/Association for Molecular Pathology; AF—allele frequency; CADD—combined annotation dependent depletion; gnomAD—Genome Aggregation Database; HGVSc—Human Genome Variation Society coding sequence name; HGVSp—Human Genome Variation Society protein sequence name; LP—likely pathogenic; VUS—variant of uncertain significance.
