# Supplementary material for: Genetic Determinants of Severe Hypertriglyceridemia: Rare Variants in LPL, APOC2, APOA5, GPIHBP1, LMF1, APOE and Polygenic Risk
Source: Int J Mol Sci. 2026 Jun 16;27(12):5443. doi: 10.3390/ijms27125443 (PMC13300099; doi:10.3390/ijms27125443)
Supplement: Supplementary file 1 [file ijms-27-05443-s001.zip › TableS1_Supplementary Material.pdf]

Supplementary Table S1. Genetic and clinical characteristics of individuals carrying rare variants in chylomicronemia genes and/or *APOE*, including the ε2/ε2 genotype

Carrier ID	Gene	HGVSp/HGVSc	Zygosity	ACMG Class	*APOE* Genotype	TG PRS, Percentile	Genetic group	Sex	Age, Years	Glucose metabolism disorders	Metabolic syndrome components	Pancreatitis	CAD	Cutaneous xanthomas	Maximal TG, mmol/L
Carriers of variants in chylomicronemia genes, including two patients with additional rare *APOE* variants and two with the *APOE* ε2/ε2 genotype															
1	*LPL*	p.Lys40AsnfsTer4	homozygote	P	ε3/ε3	84	FCS	F	28	no	no	ND	no	ND	41.0
	APOA5	p.Arg233Gln	heterozygote	VUS											
2	*LPL*	p.Trp113Arg	compound heterozygote	P	ε3/ε4	89	MCS	F	40	no	yes	no	no	no	17.31
		p.Gly59Arg		VUS											
3	*LPL*	p.Trp91Gly	heterozygote	VUS	ε3/ε3	3	MCS	M	47	no	no	no	yes	no	5.01 [1]
4	*LPL*	p.Trp113Arg	heterozygote	P	ε3/ε3	26	MCS	M	52	no	no	no	no	no	12.02
5	*LPL*	p.Trp113Arg	heterozygote	P	ε3/ε3	42	MCS	M	47	no	yes	no	no	no	7.69 [1]
6	*LPL*	p.Pro187Arg	compound heterozygote	VUS	ε3/ε3	92	MCS	M	55	no	no	yes	no	no	22.30
		p.His342Tyr													
7	*LPL*	p.Asp202Asn	compound heterozygote	LP	ε3/ε3	69	FCS	F	42	yes	no	yes	no	no	26.28
		p.Tyr233Cys													
	APOE	p.Arg154Cys	heterozygote												
8	*LPL*	p.Asp202Asn	heterozygote	LP	ε2/ε2	42	FD	F	50	yes	yes	yes	no	no	25.0 [1]
9	*LPL*	p.Gly215Glu	homozygote	P	ε3/ε4	25	FCS	M	46	yes	no	yes	yes	—	34.31
10	*LPL*	p.Gly215Glu	heterozygote	P	ε2/ε3	33	MCS	M	65	no	no	yes	no	no	24.74
11	*LPL*	p.Ala248Pro	heterozygote	VUS	ε3/ε3	75	MCS	M	54	yes	no	yes	no	no	25.16
	APOE	p.Thr307Ile	heterozygote	VUS											
12	*LPL*	p.Ile252Thr	heterozygote	P	ε2/ε4	97	MCS	M	41	no	no	no	no	no	21.33
13	*LPL*	p.His273Arg	double heterozygote	LP	ε2/ε4	69	MCS	M	49	yes	no	ND	no	ND	18.40
	APOA5	p.Gly185Cys		VUS											
14	*LPL*	p.Leu392Ter	double heterozygote	P	ε3/ε3	27	FCS	F	49	yes	yes	yes	no	no	30.59 [1]
	GPIHBP1	p.Gly175Arg		LP											
15	*LPL*	2-kb duplication	compound heterozygote	P	ε3/ε4	10	FCS	F	53	no	no	yes	no	no	47.89
		c.1019-3C>A		LP											
16	*APOA5*	p.Gln97Ter	homozygote	P	ε3/ε3	62	FCS	F	62	no	no	no	no	no	19.92 [1]
17	*APOA5*	p.Tyr194GlyfsTer69	heterozygote	P	ε3/ε3	99	MCS	M	44	no	yes	no	no	no	29.31 [1]
18	*APOA5*	p.Gly185Cys	heterozygote	VUS	ε3/ε3	70	MCS	M	63	no	no	no	no	no	15.30
19	*APOA5*	p.Gly185Cys	heterozygote	VUS	ε3/ε3	76	MCS	M	38	no	yes	no	no	ND	8.27
20	*APOA5*	p.Gly185Cys	heterozygote	VUS	ε4/ε4	44	MCS	M	43	yes	no	no	no	no	23.18 [1]
21	*APOA5*	p.Gly185Cys	heterozygote	VUS	ε3/ε4	86	MCS	F	34	yes	no	no	no	yes	92.40
22	*APOA5*	p.Gly185Cys	heterozygote	VUS	ε3/ε4	81	MCS	F	65	yes	yes	no	yes	no	5.85 [1]
23	*APOA5*	p.Gly203Arg	heterozygote	VUS	ε2/ε3	93	MCS	F	62	yes	no	no	no	no	32.0
24	*APOA5*	p.Arg223Cys	heterozygote	VUS	ε2/ε3	99	MCS	F	48	no	no	no	no	no	32.0

25	*GPIHBP1*	p.Gly123Glu	heterozygote	VUS	ε3/ε3	97	MCS	F	81	yes	no	no	no	no	6.13 [1]
26	*GPIHBP1*	p.Glu162Lys	heterozygote	VUS	ε3/ε3	74	MCS	M	36	yes	yes	no	yes	no	6.50
27	*APOC2*	p.Leu37SerfsTer4	double heterozygote	P	ε2/ε3	84	MCS	M	51	yes	no	yes	yes	yes	21.39 [1]
	LMF1	p.Met148Lys		VUS											
28	*APOC2*	p.Lys41Thr	heterozygote	VUS	ε3/ε3	65	MCS	M	33	no	no	no	no	no	6.0
29	*LMF1*	p.Tyr439Cys	heterozygote	LP	ε2/ε2	54	FD	M	41	no	no	no	no	no	9.26
30	*LMF1*	p.Tyr439Cys	homozygote	LP	ε3/ε3	18	FCS	M	32	no	no	yes	no	no	27.36 [1]
	APOE	p.Gly200Ala	compound heterozygote	VUS											
	APOE	p.Pro201Ser		VUS											
Carriers of rare *APOE* variants not overlapping with chylomicronemia gene variants															
31	*APOE*	p.Gly145Asp	heterozygote	LP	ε2/ε1	93	FD	M	40	yes	yes	no	yes	yes	12.45 [1]
32		p.Gly145Asp	heterozygote	LP	ε2/ε1	85	FD	M	37	no	no	no	no	yes	9.60
33		p.Gly145Asp	heterozygote	LP	ε2/ε1	52	FD	M	53	no	no	no	no	yes	9.87
34		p.Arg154Cys	heterozygote	LP	ε3/ε3	92	FD	M	43	no	yes	no	no	no	14.11
35		p.Arg154Cys	heterozygote	LP	ε3/ε3	76	FD	M	30	no	no	no	no	yes	9.51
36		p.Arg154Cys	heterozygote	LP	ε3/ε3	58	FD	F	58	yes	no	no	no	no	19.31
37		p.Arg154Cys	heterozygote	LP	ε3/ε3	84	FD	F	55	no	no	no	no	no	10.49
38		p.Pro201Ser	heterozygote	VUS	ε4/ε4	90	FD	M	49	no	no	no	no	no	15.50
39		p.Glu230Lys	heterozygote	LP	ε3/ε3	51	FD	M	50	yes	yes	no	yes	no	5.26
ε2/ε2 *APOE* genotype carriers only															
40	*APOE*	p.Arg176Cys	homozygote	Pathogenic for FD	ε2/ε2	89	FD	M	49	no	no	no	no	no	6.30
41						61	FD	M	37	yes	yes	no	yes	no	5.94 [1]
42						94	FD	F	27	no	yes	no	yes	no	8.41 [1]
43						88	FD	M	47	no	no	no	no	no	7.52 [1]
44						100	FD	F	42	no	no	no	no	no	13.67
45						86	FD	F	52	no	no	no	no	yes	13.12
46						21	FD	F	63	yes	no	yes	no	ND	15.32
47						100	FD	F	36	no	no	no	no	no	11.02
48						95	FD	F	66	no	yes	no	yes	no	5.31

[1] In 15 patients, maximal TG levels were available only on lipid-lowering therapy and were not recalculated. ACMG—American College of Medical Genetics and Genomics/Association for Molecular Pathology; CAD—coronary artery disease; FCS—familial chylomicronemia syndrome; FD—familial dysbetalipoproteinemia; HGVSc—Human Genome Variation Society coding sequence name; HGVSp—Human Genome Variation Society protein; LP—likely pathogenic; MCS—multifactorial chylomicronemia syndrome; ND—no data; P—pathogenic; PRS—polygenic risk score; TG— triglycerides; VUS—variant of uncertain significance.
