# Supplementary material for: Genetic Determinants of Severe Hypertriglyceridemia: Rare Variants in LPL, APOC2, APOA5, GPIHBP1, LMF1, APOE and Polygenic Risk
Source: Int J Mol Sci. 2026 Jun 16;27(12):5443. doi: 10.3390/ijms27125443 (PMC13300099; doi:10.3390/ijms27125443)
Supplement: Supplementary file 1 [file ijms-27-05443-s001.zip › TableS2_Supplementary Material.pdf]

Supplementary Table S2. Pairwise comparison of TG levels, BMI, the risk of pancreatitis, and PRS percentiles between genetic groups.

Comparison, TG levels	Estimate (log_2 TG)	Fold-change (mmol/L)	95% CI	*p*, covariate-adjusted[1]	*p*, Holm-Bonferroni adjusted[2]
**Across five groups**					
FCS vs. Moderate-to-low PRS	1.0	2.01	0.56–1.45	<0.001	<0.001
FCS vs. Polygenic HTG	0.68	1.60	0.19–1.17	0.007	0.046
FCS vs. MCS	0.80	1.75	0.15–1.46	0.016	0.080
FCS vs. FD	1.50	2.82	1.05–1.95	<0.001	<0.001
Moderate-to-low PRS vs. Polygenic HTG	-0.32	0.80	-0.70; 0.06	0.095	0.286
Moderate-to-low PRS vs. MCS	-0.20	0.87	-0.78; 0.38	0.495	0.990
Moderate-to-low PRS vs. FD	0.49	1.41	0.11–0.88	0.012	0.071
MCS vs. Polygenic HTG	-0.12	0.92	-0.69; 0.45	0.670	0.990
FD vs. Polygenic HTG	-0.82	0.57	-1.22; -0.42	<0.001	<0.001
FD vs. MCS	-0.69	0.62	-1.28; -0.11	0.020	0.81

Pairwise comparison of TG levels. Estimates represent differences in estimated marginal means from robust linear models on log_2-transformed TG levels, adjusted for sex, age, and lipid-lowering therapy. Fold-change was calculated as 2^(Estimate). [1] *p*-values (uncorrected) correspond to covariate-adjusted model estimates. [2] *p*-values adjusted for multiple comparisons using the Holm–Bonferroni method. CI—confidence interval; FCS—familial chylomicronemia syndrome; FD—familial dysbetalipoproteinemia; HTG—hypertriglyceridemia; MCS—multifactorial familial chylomicronemia syndrome; PRS—polygenic risk score.

Comparison, BMI, kg/m²	Estimate (kg/m²)	95% CI	*p*, covariate-adjusted[1]	*p*, Holm-Bonferroni adjusted[2]
**Across five groups**				
FCS vs. Moderate-to-low PRS	-6.8	-11.1; -2.6	0.002	0.017
FCS vs. Polygenic HTG	-5.7	-9.9; -1.5	0.008	0.073
FCS vs. MCS	-3.8	-8.4; 0.8	0.106	0.636
FCS vs. FD	-5.6	-10.0; -1.3	0.012	0.093
Moderate-to-low PRS vs. Polygenic HTG	1.1	-1.1; 3.4	0.325	0.974
Moderate-to-low PRS vs. MCS	3.0	0.1–5.9	0.040	0.277
Moderate-to-low PRS vs. FD	1.2	-1.3; 3.8	0.346	0.974
MCS vs. Polygenic HTG	1.9	-0.8; 4.6	0.159	0.796
FD vs. Polygenic HTG	-0.1	-2.5; 2.3	0.939	0.974
FD vs. MCS	1.8	-1.2; 4.8	0.232	0.930

Pairwise comparison of BMI. Estimates represent differences in estimated marginal means of BMI (kg/m²), adjusted for sex and age. [1] *p*-values (uncorrected) correspond to covariate-adjusted model estimates. [2] *p*-values adjusted for multiple comparisons using the Holm–Bonferroni method. BMI—body mass index; CI—confidence interval; FCS—familial chylomicronemia syndrome; FD—familial dysbetalipoproteinemia; HTG—hypertriglyceridemia; MCS—multifactorial familial chylomicronemia syndrome; PRS—polygenic risk score.

Comparison, the risk of pancreatitis	OR	95% CI	*p*, covariate-adjusted[1]	*p*, Holm-Bonferroni adjusted[2]
**Across five groups**				
FCS vs. Moderate-to-low PRS	6.24	1.06–66.3	0.042	0.253
FCS vs. Polygenic HTG	11.30	1.77–128.0	0.009	0.056
FCS vs. MCS	10.0	1.43–122.0	0.019	0.116
FCS vs. FD	24.20	3.12–338.0	0.002	0.01
Moderate-to-low PRS vs. Polygenic HTG	1.81	0.62–5.70	0.280	1.0
Moderate-to-low PRS vs. MCS	1.61	0.48–6.18	0.452	1.0
Moderate-to-low PRS vs. FD	3.88	1.0–21.7	0.05	0.251
MCS vs. Polygenic HTG	1.13	0.27–4.39	0.864	1.0
FD vs. Polygenic HTG	0.47	0.08–2.11	0.333	1.0
FD vs. MCS	0.42	0.06–2.20	0.303	1.0

Pairwise comparison of pancreatitis risk. OR and 95% CI were estimated using Firth penalized logistic regression adjusted for sex and age. Pairwise comparisons were obtained within the same model framework by varying the reference category.[1] *p*-values are based on Firth profile likelihood tests from the covariate-adjusted model. [2] *p*-values adjusted for multiple comparisons using the Holm–Bonferroni method. CI—confidence interval; OR—odds ratio; FCS—familial chylomicronemia syndrome; FD—familial dysbetalipoproteinemia; HTG—hypertriglyceridemia; MCS—multifactorial familial chylomicronemia syndrome; PRS—polygenic risk score.

Comparison, PRS percentiles	ΔMe	95% CI	*p*, unadjusted[1]	*p*, Holm-Bonferroni adjusted[2]
**Across four groups**				
FCS vs. Moderate-to-low PRS	−25	−58; −4	0.015	0.078
FCS vs. MCS	−30	−62; −6	0.020	0.078
FCS vs. FD	−32	−66; −10	0.013	0.078
FD vs. Moderate-to-low PRS	8	0–15	0.048	0.144
FD vs. MCS	3	−10; 16.0	0.602	0.788
MCS vs. Moderate-to-low PRS	5	−7; 13	0.394	0.788

Pairwise comparison of PRS distribution. Pairwise comparisons were performed using the Mann–Whitney U test. ΔMe and 95% CI were estimated using the Hodges–Lehmann method.
[1] Unadjusted *p*-values. [2] *p*-values adjusted for multiple comparisons using the Holm–Bonferroni method. ΔMe—median differences; CI—confidence interval; FCS—familial chylomicronemia syndrome; FD—familial dysbetalipoproteinemia; MCS—multifactorial familial chylomicronemia syndrome; PRS—polygenic risk scores.
